# What is your diagnosis?

**DOI:** 10.4274/jtgga.2017.0023

**Published:** 2017-09-01

**Authors:** Kavita Khoiwal, V. Seenu, Neena Malhotra, K. Aparna Sharma, Sandeep Mathur

**Affiliations:** 1 Department of Obstetrics and Gynecology, All India Institute of Medical Sciences, New Delhi, India; 2 Department of Surgery, All India Institute of Medical Sciences, New Delhi, India; 3 Department of Pathology, All India Institute of Medical Sciences, New Delhi, India

A P2L2 woman aged 35 years presented to our outpatient department with amenorrhea of 8 months with episodes of on-and-off heavy bleeding per vaginum. She had a history of laparoscopic right salpingectomy for right tubal ectopic pregnancy 8 months previously, which was confirmed on histopathology (HPE). When she presented to us, she was carrying ultrasonography (USG) of her pelvis, computed tomography (CT) of the abdomen and pelvis, and several human chorionic gonadotropin (beta-hCG) reports of last 8 months. Imaging was absolutely normal and beta-hCG values were showing a waxing and waning course (Figure 1), but never came below a discriminatory zone despite two courses of methotrexate (MTX) in doses of 50 mg/m2 in single dose regimens. A provisional diagnosis of pregnancy of unknown location (PUL) was made at that time because a pregnancy test was positive, serum beta-hCG was 10,304 IU/L, and no intra or extra-uterine gestation could be cited on ultrasound. She had no spells of abdominal pain or fainting attacks. On being referred to our unit, we evaluated her on the lines of gestational trophoblastic neoplasia (GTN) in view of persistently high beta-hCG for the last 8 months (graphical presentation of beta-hCG shown in Figure 1. Routine blood investigations, chest X-ray, USG pelvis, as well as CT chest and brain were found as normal. We did not repeat CT of the abdomen and pelvis. Considering persistently high beta-hCG values without any evidence of pregnancy on imaging, two courses of single agent chemotherapy (MTX-FA) in a multiple dosage regimen (Inj. MTX 1 mg/kg/day on days 1, 3, 5, and 7, and Inj. Folinic acid 15 mg on day 2, 4, 6, and 8) was given intravenously, 2 weeks apart; however, beta-hCG value remained high.

## ANSWER

**Figure 1:**
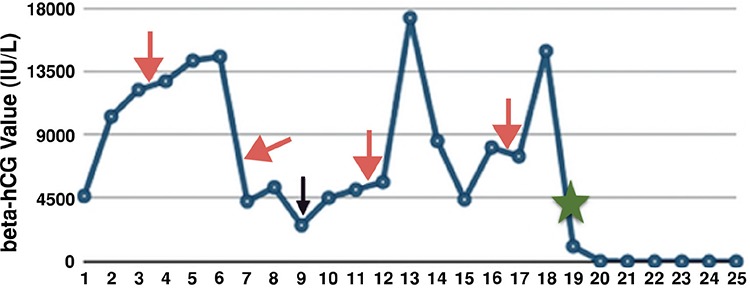
Graphical presentation of beta-human chorionic gonadotropin values (red arrow indicates the timing of single-agent chemotherapy, the purple arrow shows the time of referral to us, and the green star is the time of surgery)

**Figure 2:**
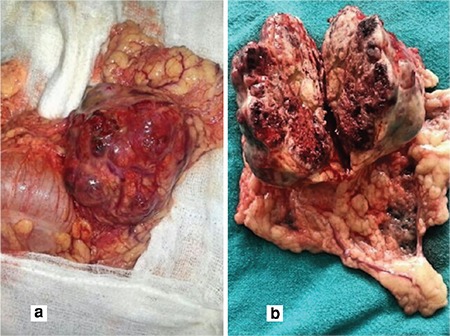
(a). A 5x5-cm firm, well circumscribed and encapsulated mass over the greater omentum (b). Cut section of the mass showing extensive areas of necrosis and hemorrhage

**Figure 3:**
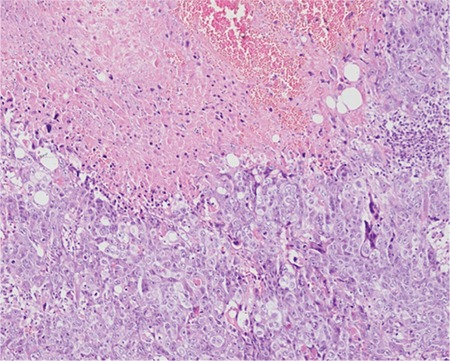
H&E (x100) stained section showing necrotic area along with the tumor

**Figure 4:**
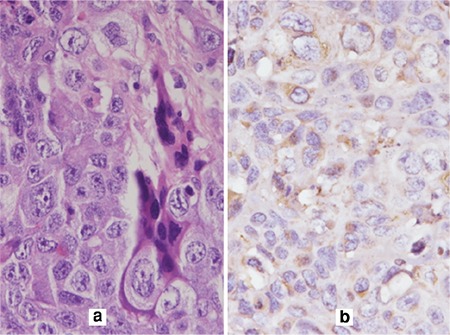
(a). H&E (x400) showing biphasic tumor comprising of giant cells and mononuclear tumor cells, (b). IHC-Positive for beta-human chorionic gonadotropin

After a thorough discussion and failure to find the source of beta-hCG, the decision was taken to perform a whole-body positron emission tomography (PET)-CT scan. On PET-CT, an active 5x4-cm malignant lesion was found in the bowel mesentery. No metastasis was found in any other site. The patient underwent exploratory laparotomy. Intra-operatively, a 5x5-cm, firm, well-circumscribed and encapsulated mass was present over the greater omentum, as shown in Figure 2a. The whole mass, with a 2-cm healthy omental margin, was excised. The cut section of the mass showed extensive areas of necrosis and hemorrhage (Figure 2b). The uterus and bilateral adnexa were healthy. The right fallopian tube was found absent intra-operatively. The mass was confirmed as an omental choriocarcinoma on HPE (Figure 3-4). Postoperatively, her beta-hCG value decreased dramatically to 1073 IU/L, and 112 IU/L after one week. She required no adjuvant chemotherapy because there was no evidence of local or distant metastasis. The patient was under regular follow-up with us as well as the medical oncology department. Beta-hCG follow-up was performed weekly until three negative values were obtained, and monthly thereafter for 24 months. During follow-up, her beta-hCG value always remained negative (Figure 1). She never had any signs and symptoms of recurrence. The patient is now disease free.

**Figure 5:**
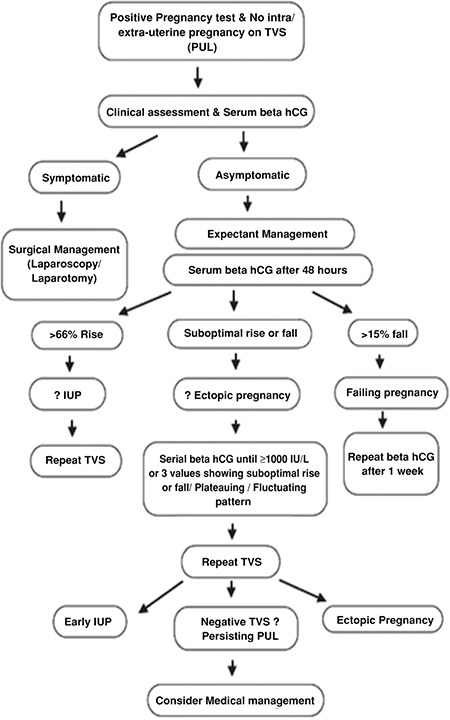
An algorithm for the management of pregnancy of unknown location *IUP: intra-uterine pregnancy; TVS: trans vaginal scan*

PUL is defined by the presence of a positive pregnancy test with an absence of either intra or extrauterine pregnancy on transvaginal ultrasound (1). The reported incidence of PUL is 8-10% (1). MTX 50 mg/m^2^ has proven to be effective in 90% of cases of ectopic pregnancy and also results in resolution of beta-hCG levels in women with asymptomatic persisting PUL (1). An algorithm for the management of PUL is described in Figure 5 (1).

GTN usually occurs after molar pregnancy (50%), but may also be present after abortions (25%), normal intrauterine pregnancy (22.5%), and ectopic pregnancy (2.5%) (2). Choriocarcinoma per se is a rare tumor. The incidence of choriocarcinoma is 1 in 45,000 pregnancies in Western countries, but a higher incidence is reported from southeast Asia (3). Choriocarcinoma is a malignant and aggressive tumor, associated with high beta-hCG. Most cases of choriocarcinoma are intrauterine and of gestational origin. Extrauterine choriocarcinoma is quite rare, few case reports are present in the literature (4, 5, 6, 7, 8, 9, 10, 11, 12). Moreover, choriocarcinoma of the greater omentum is extremely rare, with only two cited cases (8, 11). Most cases developed secondary to implantation of trophoblastic tissue following an ectopic gestation (4, 5, 6, 7, 8) and sited in the genital tract (tube, ovary, cervix, and vagina) (4, 5, 6, 7). A case of omental choriocarcinoma mimicking ectopic pregnancy was reported by Wan et al. (8). Their patient required multiple courses of combined chemotherapy to achieve remission. The plausible etiology of this lesion in our case was the persistence of trophoblastic tissue left during laparoscopic salpingectomy that may have got implanted over the omentum. In the case of primary extrauterine choriocarcinoma, the uterus, tubes, and ovaries show no evidence of pregnancy. On reviewing the literature, four cases of primary choriocarcinoma could be elucidated occurring on the left anterior abdominal wall of the pelvis (9), on the surface of a subserosal uterine leiomyoma (10), in the omentum (11), and the right lower lobe of the lung (12). All these cases were managed by complete resection of tumor ± adjuvant chemotherapy. The prognosis of such cases is not well studied due to its rarity, but according to the above case reports, most patients achieved complete remission.

We report this case of choriocarcinoma of the greater omentum not for its rarity, but for the fact that it was missed despite an intensive examination. Our patient was initially managed along the lines of PUL considering that imaging did not support any ectopic site for the source of beta hCG. That an initial baseline CT showed no tumor goes with the understanding that perhaps the lesion was small enough to be missed on CT, yet it produced enough beta hCG, and was uncompromising to chemotherapy. The lesion may have increased in size because no imaging was repeated during MTX cycles. We decided to perform a PET-CT for the whole body in the absence of an adequate response to standard chemotherapy, which was the turning point in our case because we established the source of beta-hCG. The efficacy of PET-CT in defining such lesions is not known, perhaps due to the paucity of such cases, which typically undergo surgery in good time with a provisional diagnosis of ectopic pregnancy.

## CONCLUSION

Rare sites of choriocarcinoma can be missed despite intensive examinations. A suspicion of disease should always be kept when serial beta hCG levels show a plateau or rising trends despite chemotherapy. PET-CT may be a useful adjunct to map such lesions. Surgical excision of these tumors remains the best modality in resectable cases. Close follow-up is mandatory to achieve complete remission.
